# Artificial intelligence in the diagnosis and prognosis of ocular trauma: a systematic review

**DOI:** 10.1186/s12886-026-04688-x

**Published:** 2026-03-14

**Authors:** Zahra Abbasi Dolatabadi, Mahdi Nabi Foodani, Mohammad Fayyazi Farkhad, Faezeh Golvardi-Yazdi

**Affiliations:** 1 Department of Medical Surgical Nursing, School of Nursing and Midwifery, Tehran University of Medical Sciences, Tehran, Iran; 2 Road Traffic Injury Research Center, Tabriz University of Medical Sciences, Tabriz, Iran; 3 Department of Emergency Nursing, School of Nursing and Midwifery, Tehran University of Medical Sciences, Tehran, 1419733171 Iran

**Keywords:** Systematic review, Prognosis, Diagnosis, Eye injuries, Artificial intelligence

## Abstract

**Background:**

Ocular trauma is a leading cause of acquired monocular blindness worldwide, requiring prompt and accurate diagnosis and prognosis. While artificial intelligence (AI) has shown growing potential in medicine, a comprehensive synthesis of its applications in Diagnosis and Prognosis of Ocular Trauma is still lacking. Therefore, this systematic review aims to comprehensively synthesize the current evidence on the diagnostic and prognostic performance of artificial intelligence in ocular trauma.

**Methods:**

This study is a systematic review conducted in accordance with the PRISMA 2020 guidelines. A comprehensive search was performed in PubMed, Scopus, and Web of Science from inception to May, 2025. Original studies using artificial intelligence (AI) for the diagnosis or prognosis of ocular trauma, applying human clinical data, and reporting performance metrics were included. Review articles and animal studies were excluded. Two reviewers independently screened the studies, extracted relevant data, and assessed the risk of bias using the PROBAST tool. Due to substantial heterogeneity among the included studies, a meta-analysis was not performed. This heterogeneity was primarily related to marked differences in outcome definitions, study designs, analytical models, and reported effect measures across studies. Specifically, the included studies employed diverse outcome metrics, used varying model types, and analyzed different data modalities, with effect estimates reported in non-comparable forms. As a result, quantitative pooling of data was not methodologically appropriate. Therefore, the findings were synthesized using a narrative approach. Heterogeneity was explored descriptively by examining the variability in reported AUC ranges across relevant subgroups, including outcome definitions, model types, and data modalities. Formal quantitative heterogeneity statistics were not calculated due to inconsistent reporting and insufficient data within subgroups.

**Results:**

A total of 112 records were identified across PubMed, Scopus, and Web of Science. After removing duplicates and screening titles and abstracts, 10 studies met the predefined inclusion criteria and were systematically analyzed. These studies investigated different AI techniques applied to ocular trauma. Across the included studies, deep learning models such as DenseNet-169 and UNet demonstrated high diagnostic accuracy (up to 96%) and Area under the curve (AUC) values of 0.99, while artificial neural networks (ANNs) outperformed traditional scoring systems like the Ocular Trauma Score (OTS) in prognostic prediction (accuracy up to 93%). ChatGPT-4 showed perfect diagnostic accuracy (100%) but a 30% inconsistency in treatment decisions. A narrative synthesis revealed that model performance varied by input data type, with image-based models generally outperforming those relying on clinical data alone.

**Conclusions:**

The findings suggest that AI models, particularly deep learning and neural network architectures, can assist clinicians in rapid and accurate diagnosis, optimize triage decisions in emergency settings, and improve prognostic prediction of visual outcomes. Despite these promising results, challenges such as variability in data sources, lack of external validation, and ethical considerations persist. Future research should focus on multicenter validation, standardized datasets, and human–AI integration frameworks to facilitate clinical application.

**PROSPERO registration code:**

CRD420251043768

## Introduction

Ocular trauma represents one of the most frequent and vision-threatening emergencies encountered in ophthalmic and emergency care settings. It remains the leading cause of acquired monocular blindness worldwide, affecting both children and adults across diverse geographical and socioeconomic contexts [1, 2]. Such injuries arise from a wide range of mechanisms—including blunt and penetrating trauma, intraocular foreign body, chemical or thermal exposure, falls, assaults, and motor vehicle accidents—and present with high heterogeneity in severity and clinical manifestations. The urgent nature of these injuries, combined with the complexity of ocular anatomy and potential for rapid deterioration, underscores the importance of prompt and accurate diagnosis to avoid irreversible vision loss [3, 4].

Although standard clinical guidelines exist for the management of ocular trauma [5–7], delays in diagnosis or misinterpretation of key findings may critically compromise functional outcomes. Globally, more than 19 million individuals are estimated to experience monocular blindness due to ocular injuries, reflecting the substantial healthcare and socioeconomic burden associated with traumatic eye conditions [8–10]. Globally, approximately 19 million individuals suffer from monocular blindness caused by traumatic eye injuries, which poses a heavy burden on public health systems because of both high treatment costs and socioeconomic consequences [9, 10].

Parallel to the growing clinical challenges associated with ocular trauma, the field of ophthalmology has undergone rapid transformation driven by advances in artificial intelligence (AI). Since its conceptual introduction by John McCarthy in 1956, AI has evolved from basic rule-based systems to sophisticated machine learning (ML) and deep learning (DL) models capable of processing complex datasets, identifying intricate patterns, and generating predictive outputs with remarkable consistency and accuracy [11–13].

These techniques have been widely adopted in ophthalmic diagnostics, particularly for automated detection of diabetic retinopathy, age-related macular degeneration, retinal detachment, and glaucoma progression, often demonstrating expert-level or superior performance across various imaging modalities [14, 15].

Compared with other ophthalmic diseases, the application of AI in ocular trauma is relatively recent but expanding rapidly [16]. Traumatic injuries pose unique diagnostic challenges due to obscured visualization, concurrent hemorrhage or edema, and the need for rapid interpretation of imaging studies such as computed tomography (CT). In recent years, diverse AI models—including convolutional neural networks for orbital fracture detection and segmentation [17], ML-based algorithms for predicting visual outcomes [18], and natural language processing (NLP) tools and large language models (LLMs) for clinical reasoning—have been developed to enhance both diagnostic precision and prognostic accuracy in ocular trauma cases [14]. Studies applying deep convolutional neural networks to orbital CT imaging report high classification accuracy, often approaching or exceeding traditional observer performance in fracture detection tasks [17]. Furthermore, emerging studies evaluating LLMs like ChatGPT demonstrate promising capability in diagnostic reasoning and triage support, though inconsistencies remain in treatment decision-making [14].

Despite these advances, the current body of evidence remains fragmented. Studies vary substantially in their methodological quality, data sources, AI techniques, performance metrics, and clinical endpoints. Moreover, no previous systematic review has provided a comprehensive synthesis of diagnostic and prognostic performance across AI models used in ocular trauma. As a result, clinicians, researchers, and policymakers lack clear and consolidated evidence regarding the reliability, clinical utility, and limitations of AI-based tools in this domain.

To address this gap, the present systematic review critically synthesizes the existing evidence on artificial intelligence–based models applied to ocular trauma. Specifically, this review aims to [1] characterize the range of AI methodologies employed for diagnostic and prognostic tasks [2], summarize reported model performance across different trauma types and data modalities without direct quantitative comparison, and [3] identify key methodological limitations, challenges, and opportunities for future development and clinical integration. By providing a comprehensive and descriptive evidence synthesis, this review seeks to support informed decision-making regarding the potential adoption of AI-based tools in emergency and ophthalmic trauma care, while highlighting priorities for advancing research and clinical translation.

## Methods

This study is a systematic review conducted in accordance with the PRISMA 2020 guidelinesA comprehensive search was performed in PubMed, Scopus, and Web of Science between April and May 2025, with no restriction on publication date.Original studies using artificial intelligence (AI) for the diagnosis or prognosis of ocular trauma, applying human clinical data, and reporting performance metrics (e.g., accuracy, sensitivity, specificity) were included. Review articles and animal studies data were excluded. Two reviewers independently screened the studies, extracted relevant data, and assessed the risk of bias using The Prediction model Risk Of Bias ASsessment Tool (PROBAST). Due to heterogeneity among the included studies, no meta-analysis was conducted, and results were synthesized narratively.

The protocol of this review was registered in the International Prospective Register of Systematic Reviews as CRD420251043768 (https://www.crd.york.ac.uk/PROSPERO/view/CRD420251043768.) and conducted in accordance with the Preferred Reporting Items for Systematic Reviews and Meta-Analyses (PRISMA) 2020 guidelines [19]. A completed PRISMA checklist is provided in Appendix Table 1 to ensure comprehensive and transparent reporting of the review process.

### Selection criteria

Studies were included if they applied artificial intelligence for the diagnosis or prognosis of ocular trauma, used human clinical data, reported model performance metrics such as accuracy, sensitivity, specificity, or area under the curve (AUC), were published in English, and had full-text available. Studies were excluded if they were review articles, used only animal or in vitro data.

### Search strategy

The search strategy combined relevant keywords and controlled vocabulary terms related to ocular trauma, artificial intelligence, and clinical outcomes. The final search string was as follows:

(“ocular trauma” OR “eye trauma” OR “ocular injur*” OR “eye injur*” OR “close* globe injur*” OR “open* globe injur*” OR “orbital fracture” OR “chemical burn) AND (“artificial intelligence” OR “machine learning” OR “deep learning” OR “AI” OR “neural network”) AND (“diagnosis” OR “prognosis” OR “prediction” OR “predictive model” OR “triage” OR “classification”). Table 1.

**Table 1:** Search strategy

No.	Database	Results
1	PubMed	32
2	Web of Science	42
3	Scopus	38
Total	PubMed, WOS and Scopus	112
Duplicates	PubMed, WOS and Scopus	46
Total	PubMed, WOS and Scopus	66
	**PubMed: National Library of Medicine**	
#	Query	Results
1	(“ocular trauma” OR “eye trauma” OR “ocular injur*” OR “eye injur*” OR “close* globe injur*” OR “open* globe injur*” OR “orbital fracture” OR “chemical burn”)	21,600
2	(“artificial intelligence” OR “machine learning” OR “deep learning” OR “AI” OR “neural network”)	817,497
3	(“diagnosis” OR “prognosis” OR “prediction” OR “predictive model” OR “triage” OR “classification”)	6,314,465
4	#1 AND #2 AND #3	32
	**Web of Science Core Collection**	
#	Query	Results
1	(((((((ALL=(ocular trauma)) OR ALL=(eye trauma)) OR ALL=(ocular injur*)) OR ALL=(eye injur*)) OR ALL=(close* globe injur*)) OR ALL=(open* globe injur*)) OR ALL=(orbital fracture)) OR ALL=(chemical burn)	9,985
2	((((ALL=(artificial intelligence)) OR ALL=(machine learning)) OR ALL=(deep learning)) OR ALL=(AI)) OR ALL=(neural network)	2,326,818
3	(((((ALL=(diagnosis)) OR ALL=(prognosis)) OR ALL=(prediction)) OR ALL=(predictive model)) OR ALL=(triage)) OR ALL=(classification))	5,623,820
4	#1 AND #2 AND #3	38
	Scopus	
#	Query	Results
1	TITLE-ABS-KEY(“ocular trauma” OR “eye trauma” OR “ocular injur*” OR “eye injur*” OR “close* globe injur*” OR “open* globe injur*” OR “orbital fracture” OR “chemical burn”)	40,202
2	TITLE-ABS-KEY(“artificial intelligence” OR “machine learning” OR “deep learning” OR “AI” OR “neural network”)	2,574,073
3	TITLE-ABS-KEY(“diagnosis” OR “prognosis” OR “prediction” OR “predictive model” OR “triage” OR “classification”)	9,551,424
4	#1 AND #2 AND #3	57

The search strategy was tailored for each database and last updated on May 3, 2025.

### Data extraction

Data were independently extracted by two Researcher via a predesigned Excel sheet on the basis of the review protocol. The extracted information included the study characteristics, AI model type, type of ocular trauma, data source, outcome type (diagnosis or prognosis), and related metrics, such as accuracy, sensitivity, specificity, and AUC, as well as the study population, sample size, country, and model performance indicators. Disagreements were resolved through discussion.

### Data analysis

Because of the heterogeneity in study designs, AI models, and reported outcome measures, a quantitative meta-analysis was not performed. Instead, a structured narrative synthesis was conducted following PRISMA 2020 guidelines. Extracted data were analyzed descriptively according to three predefined domains aligned with the review objectives: type of ocular trauma, AI methodology, and predicted clinical outcome. Studies were organized within these categories to identify patterns and trends in AI performance. Model metrics, including accuracy, sensitivity, specificity, and AUC, were compared across subgroups, and imaging-based models were descriptively compared with those relying on clinical data to assess differences in diagnostic and prognostic accuracy. Any inconsistencies or variations across studies were qualitatively interpreted in relation to study design, data source, and risk of bias.

## Results

### Study selection

**Fig. 1:**
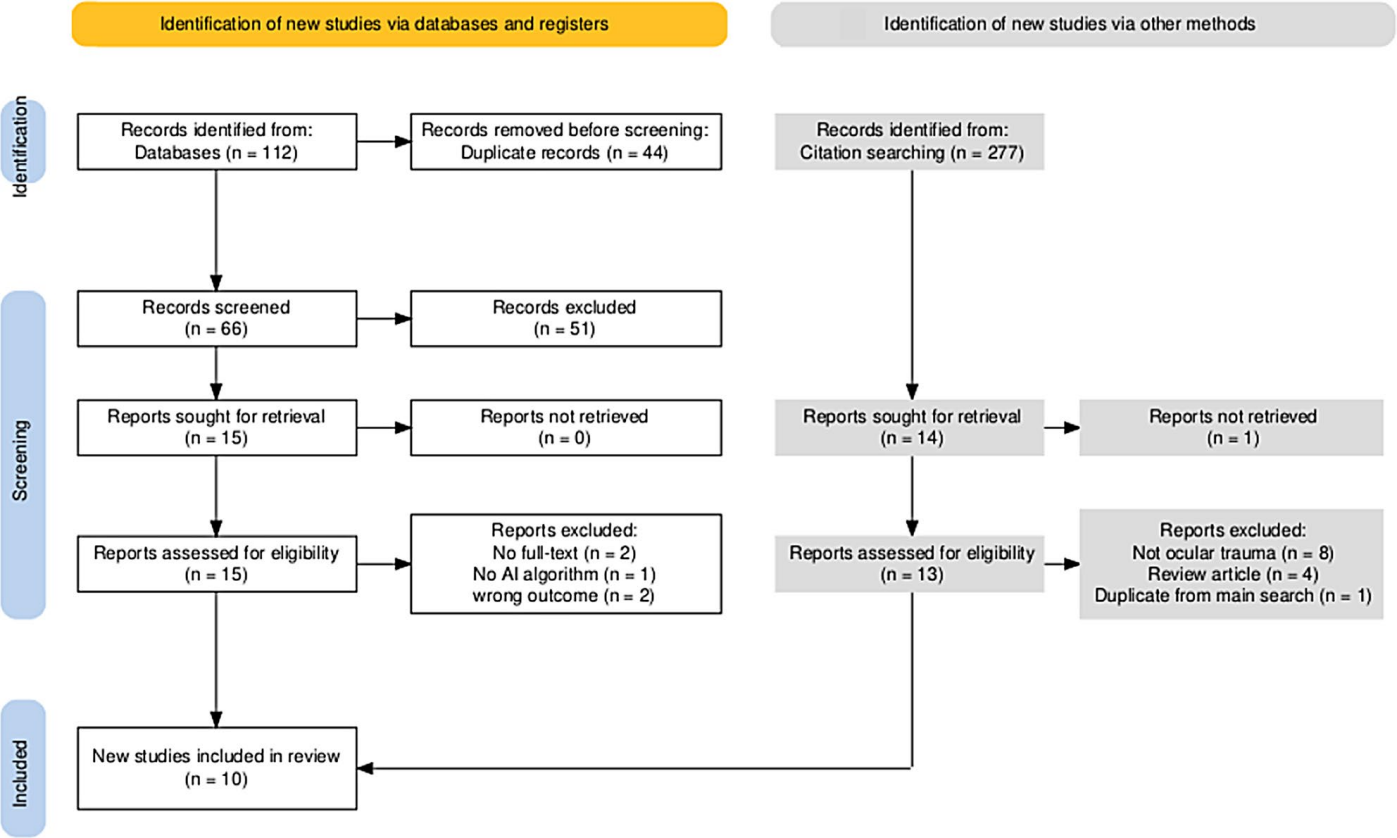
PRISMA Flow diagram of study

A comprehensive search across multiple databases identified 112 records. After 46 duplicates were removed, 66 records remained for title and abstract screening. Following this screening, 51 studies were excluded because they did not meet the inclusion criteria. The full texts of 15 articles were assessed for eligibility, and 10 studies [14, 17, 18, 20–26] met the inclusion criteria and were included in this systematic review. The study selection process is summarized in the PRISMA flow diagram is shown in Fig. 1.

### Risk of bias in studies

The risk of bias across four domains was assessed via the PROBAST tool: participants, predictors, outcomes, and analysis. Most studies showed low to moderate risk in the participant and predictor domains; however, some exhibited high risk in the outcome and analysis domains, primarily due to a lack of clarity in outcome definitions, inappropriate handling of missing data, and limited information on model overfitting and performance evaluation. The detailed evaluation of individual PROBAST questions per study is provided in Tables 2 and 3, while Table 4 summarizes the risk of bias assessments with color coding for clarity (green = low risk, yellow = moderate risk, red = high risk).

### Study characteristics

The included studies were cohort [17, 18, 21], cross-sectional [20, 22], and retrospective [14, 23–26] in design and were conducted in various countries, including the USA [23, 25], China [17, 20–22, 26], Iran [24], the Republic of Korea [18] and Switzerland [14]. A total of ten studies met the inclusion criteria, with sample sizes ranging from 100 to 3705 participants. To facilitate comparison, the included studies were categorized based on type of ocular trauma, AI methodology, and predicted clinical outcome. By trauma type, most studies focused on open-globe injuries [18, 21, 24, 25], three examined orbital fractures [14, 17, 20] and the remainder addressed mixed ocular trauma cases [22, 23, 26]. When categorized by AI methodology, deep learning–based models were predominantly used for diagnostic imaging tasks, with convolutional neural networks (CNNs) representing the most frequently employed architectures [17, 18, 20, 25]. Prognostic modeling of visual outcomes more commonly utilized traditional machine learning approaches, including artificial neural networks (ANNs) and other supervised learning algorithms [21, 24, 26]. Three recent studies [14, 22, 23] evaluated conversational AI models such as ChatGPT-4 for diagnostic reasoning and treatment decision-making. By data modality, imaging-based studies (CT or fundus photographs) [17, 20, 23, 25, 26] generally demonstrated higher diagnostic accuracy (AUC 0.90–0.99) compared to those using solely clinical features (AUC 0.78–0.85) [14, 18, 21, 22, 24]. The predicted outcomes across studies included fracture localization, segmentation of affected areas, prediction of final visual acuity, and evaluation of diagnostic or triage accuracy.A detailed, categorized summary of study characteristics is presented in Table 5.

### Study characteristics and types of AI models

The ten included studies employed a wide range of artificial intelligence (AI) approaches, thereby directly addressing the first objective of this review. Overall, the identified methods were classified into three main categories: deep learning, classical machine learning, and natural language processing with large language models (NLP/LLMs).

Deep learning approaches included architectures such as DenseNet-169, InceptionV3, MRes-UNET2D, and UNet and its modified variants. Classical machine learning methods comprised XGBoost, random and decision forests, support vector machines (SVMs), and artificial neural networks (ANNs). In addition, NLP and LLM-based techniques were represented by systems such as MedLEE and ChatGPT-4.

The heterogeneity of these AI methodologies highlights the multifaceted nature of AI applications in ocular trauma, encompassing image analysis, predictive modeling based on clinical data, extraction of relevant information from textual records, and clinical reasoning supported by large language models.

### AI performance across trauma types and data modalities

Across included studies, imaging-based deep learning models consistently demonstrated superior diagnostic performance for various ocular trauma types. DenseNet-169 achieved AUC of 0.99 and 98% accuracy in orbital fracture detection (Bao et al.) [20], while combinations of InceptionV3 and XGBoost reported AUCs of 0.957 [17]. Modified UNet architectures effectively segmented injured ocular structures, achieving Dice scores of 0.95 [25]. These results indicate that deep learning excels in detecting structural abnormalities across trauma types and image modalities, fulfilling the objective of evaluating performance across both trauma types and data modalities.

In parallel, natural language processing (NLP) and large language models (LLMs) were assessed for their diagnostic interpretation capabilities. Sarioglu et al. applied NLP to CT radiology reports and, using filtered NLP + SVM models, achieved high diagnostic accuracy with F-scores of 0.97(CI:95%) [23]. Similarly, ChatGPT-4 correctly identified all orbital fracture cases, but agreement with clinical management decisions was only 70% (Gernandt et al.) [14]. These findings demonstrate that NLP and LLMs are highly effective for extracting diagnostic information from textual reports, but therapeutic decision-making remains less reliable, highlighting modality-specific strengths and limitations.

For prognostic applications, machine learning models incorporating clinical variables showed substantial improvements over traditional scoring systems. ANN models predicted final visual acuity after ocular trauma with 93% accuracy and AUC 0.96(CI:95%) (Shariati et al.) [24], outperforming conventional tools such as the Ocular Trauma Score. Boosted decision trees yielded AUCs up to 0.97 in open globe injury prognosis (Choi et al.) [18], and the VisionGo NLP-based model achieved preoperative AUC 0.75 and intraoperative AUC 0.90, surpassing both clinician predictions and OTS benchmarks (Meng et al.) [21]. These results suggest that ML-based prognostic models, particularly those using structured clinical features, offer meaningful enhancements in outcome prediction.

### Patterns in AI performance across data types

A consistent pattern across studies was that imaging-based deep learning demonstrated superior diagnostic performance (AUC: 0.90–0.99) compared with models relying solely on clinical features (AUC: 0.78–0.85). Conversely, prognostic models incorporating clinical variables, especially those using ANN and boosted algorithms, outperformed imaging alone for predicting final visual acuity. NLP and LLM-based models showed promise for text interpretation, particularly for extracting structured information from unstructured reports.

These findings underscore the importance of matching AI model types to the clinical task:

Deep learning: best for diagnosis of structural abnormalities.

Machine learning: best for prognosis based on clinical features.

LLMs/NLP: best for decision support and textual data interpretation.

## Discussion

The findings of this study indicate that artificial intelligence (AI) has considerable potential in the diagnosis and prognostication of ocular trauma. This systematic review revealed that deep learning models such as DenseNet-169 and UNet demonstrated high performance in detecting orbital fractures, with reported AUC values reaching 0.99 and accuracies exceeding 96% in the study by Xiao-li Bao (2023) [20]. These results show that deep learning can be very helpful in improving diagnosis speed and accuracy in trauma cases.

Additionally, predictive models such as artificial neural networks (ANNs) have shown acceptable performance in stratifying final visual acuity into different levels. In the study by Shariati (2024), the ANN model achieved an accuracy of 93% and an AUC of 0.96 in predicting visual outcomes, reflecting its strong discriminative ability(CI:95%) [24]. This highlights the potential use of ANN models in early decision-making and outcome prediction in eye injuries.

However, beyond reporting high performance metrics, a critical appraisal of model validation strategies and generalizability is necessary to contextualize these findings. Most included studies primarily reported model performance based on internal validation or cross-validation, with limited evidence of external validation using independent datasets. While these approaches are useful for assessing model consistency within a given dataset, they may overestimate real-world performance when models are applied across different populations, imaging protocols, or clinical environments. External validation, which is essential for evaluating model robustness and transportability, was infrequently performed. Consequently, the high accuracy and AUC values reported in several studies should be interpreted cautiously, as their generalizability to routine clinical practice remains uncertain.

In addition, apparent high diagnostic performance may partly reflect methodological limitations, including class imbalance and an over-reliance on discrimination metrics such as AUC. Many studies did not report complementary measures of model calibration or clinical utility, such as calibration slope, Brier score, or Matthews correlation coefficient. In imbalanced datasets, high AUC values may obscure poor predictive performance for less frequent but clinically critical outcomes. The absence of calibration reporting further limits assessment of whether predicted probabilities accurately reflect observed risks, which is essential for clinical decision-making. Future AI studies in ocular trauma should therefore integrate both discrimination and calibration metrics and prioritize validation across diverse and clinically relevant subgroups.

Recent literature within the past two years reflects significant advances in artificial intelligence applications in ophthalmology. Multi-modal AI approaches that integrate multiple data types such as imaging and clinical variables have demonstrated promising performance beyond single-modality models, suggesting enhanced diagnostic and prognostic utility across diverse ocular conditions [27, 28]. In addition, large language models with visual and textual input capabilities, such as GPT-4 V, have shown potential for combined analysis of ocular images and clinical context, representing an emerging direction for AI-assisted decision support in ophthalmic practice [29]. These developments indicate that contemporary AI research in ophthalmology is rapidly evolving toward more comprehensive and robust multimodal systems.

The nature of diagnosis by humans and AI is fundamentally different. Human diagnostic performance is influenced by factors such as clinical experience, environmental conditions, and subjective interpretation, whereas AI models when properly programmed and adequately trained can interpret input data consistently and efficiently, delivering results in less time and at a broader scale. The findings suggest that the ANN model in Shariati’s study outperformed traditional tools such as the OTS. The ANN model functions independently of human intervention, relying solely on structured clinical features [24]. In contrast, the OTS system, designed primarily for binary outcome prediction (vision vs. no vision), has limited precision in multiclass predictions and is heavily reliant on clinical judgment [30, 31]. This suggests that AI models can reduce human error and offer more detailed outcome predictions compared to traditional scoring tools.

Although ultrasonography has demonstrated high diagnostic accuracy for ocular trauma when performed by experienced clinicians [32, 33], its operator-dependent nature and required expertise limit consistency across settings. AI-based imaging approaches may help address these limitations by providing more standardized and reproducible diagnostic support.

Moreover, a retrospective study by Gernandt (2023) demonstrated that ChatGPT-4 successfully diagnosed all cases of orbital fracture with 100% accuracy. However, its treatment recommendations (surgical vs. conservative) align with actual clinical decisions in only 70% of cases [14]. This means that although AI can help in diagnosis, it still needs improvement when used for making treatment decisions.

In addition to ocular trauma, recent studies have demonstrated the high efficacy of deep learning in diagnosing maxillofacial fractures. For example, Warin et al. (2023) evaluated CNN-based models including DenseNet-169, ResNet-152, YOLOv5, and Faster R-CNN on CT images. The overall accuracy of DenseNet-169 in multi-class classification is reported to be 0.7, and the accuracy of the Faster R-CNN model in identifying failure locations is reported to be 0.78 [34]. Furthermore, Dashti et al. (2024) conducted a meta-analysis on deep learning algorithms applied to panoramic radiographs for mandibular fracture detection, reporting pooled sensitivity of ~ 97% and specificity of ~ 81% [35]. These findings show that AI models used in eye injuries can also work well in detecting other types of trauma, like jaw or facial fractures.

From a comparative perspective, our review identifies clear performance differences across AI model types. Image-based deep learning models such as DenseNet and UNet demonstrated superior performance in CT-based diagnostic tasks compared to traditional machine learning approaches like logistic regression or decision trees. In terms of prognostic modeling, ANNs trained on diverse clinical variables consistently outperformed algorithms such as support vector machines and decision forests, as observed in Shariati et al. (2024) [24]. Choosing the right model based on the type of data and clinical goal is important for achieving the best results.

NLP-based models like ChatGPT-4 were highly effective in processing narrative clinical data for diagnostic hypothesis generation but showed limitations in decision-making accuracy. Importantly, model interpretability remains a key issue in clinical AI. While most high-performing deep learning models function as “black boxes,” tools like VisionGo (Meng et al., 2024) incorporated SHAP (Shapley Additive Explanations) to enhance transparency and explainability [21]. This trade-off between model complexity and interpretability is a critical factor when considering real-world deployment. AI models that are easier to understand and explain will likely be more accepted in clinical practice, especially in serious or high-risk cases.

Overall, the selection of AI models must align with the clinical objective diagnosis, prognosis, or decision support and should be guided by input data type, performance needs, and the clinical context. Imaging-based models remain most effective for detection, while clinical-feature-based ANNs offer robust outcome prediction. NLP models are best suited for triage support and case analysis but currently require further refinement for therapeutic recommendation tasks.

### Strengths and limitations

This systematic review was conducted with a rigorously designed methodology to ensure the identification and inclusion of all relevant studies. To maintain the highest standards of academic integrity, two independent reviewers systematically performed abstract screening, full-text evaluation, data extraction, and bias assessment. To mitigate potential publication bias and ensure comprehensive coverage of the literature, an extensive search strategy was implemented across multiple databases.

interpretability remains a key limitation in the clinical application of AI, as many explainability techniques such as SHAP—originally proposed as a unified framework for feature attribution in complex models [36]—are inconsistently applied across studies. Furthermore, explainability has been identified as critical for clinician trust and adoption in ophthalmic AI, since transparent reasoning enhances acceptance and safe implementation in practice [37]. In contrast, large language models such as ChatGPT-4 have recently shown potential for clinical reasoning and decision support in orbital fracture diagnosis, although domain-specific validation remains limited [14].

Despite promising findings, the evidence reviewed in this study has several limitations. Some studies had small sample sizes or relied on single-center data, which may limit the generalizability of the results. In several studies that used AI-based chatbots such as ChatGPT, there was insufficient transparency regarding the type of input data, sample selection criteria, and model testing conditions. Additionally, several studies did not assess external validity, and models were often tested in retrospective or controlled environments, limiting the generalizability of the results to real-world clinical settings.

Another important issue is the high variability in data types (including images, clinical features, and textual data) and performance metrics (such as accuracy, AUC, F1 score, and MCC), making direct comparisons between studies difficult.

The review process itself also has limitations. Some relevant studies may have been excluded due to language restrictions, unavailability of full-text articles, or the use of nonstandard terminology in databases. The lack of uniform reporting standards for AI model performance further complicated the synthesis and interpretation of the findings. Although a comprehensive search strategy was applied, the possibility of selection bias or missing unpublished research cannot be entirely ruled out.

### Implications for clinical practice, policy, and future research

This study underscores the potential of artificial intelligence in enhancing diagnostic accuracy, clinical decision-making, and prognostic prediction for patients with ocular trauma. AI-based tools may serve as valuable adjuncts to human expertise, particularly in resource-constrained settings or high-volume clinical environments, by improving diagnostic efficiency and reliability. These technologies could complement clinical judgment, especially in domains reliant on structured data or image-based assessments.

For widespread AI implementation, healthcare systems must establish robust ethical frameworks and data privacy safeguards to ensure patient information security. Future research should prioritize multicenter, real-world validation studies with larger datasets while exploring optimal human-AI collaboration models. Additionally, comparative studies evaluating AI performance against human clinicians in ocular trauma management are needed to objectively assess the clinical utility of this technology and define its optimal scope of application.

## Conclusion

The findings of this systematic review indicate that AI has significant ability in identifying the type and location of ocular fractures, predicting final visual acuity, and assisting with treatment decisions on the basis of imaging and clinical data. AI systems may closely rival, or in some instances, surpass human performance. However, the optimal clinical use of AI likely lies in its integration as a collaborative and complementary tool alongside human expertise.

## Appendix

**Table 1:** PRISMA 2020 Checklist

Section and Topic	Item #	Checklist item	Location where item is reported
TITLE			
Title	1	Identify the report as a systematic review.	Title includes “A Systematic Review” Page:1 Line:1
**ABSTRACT**			
Abstract	2	See the PRISMA 2020 for Abstracts checklist.	Structured abstract includes background, objectives, methods, results, and conclusion. Page: 2–3 Line:19–50
**INTRODUCTION**			
Rationale	3	Describe the rationale for the review in the context of existing knowledge.	Rationale is clearly discussed in the Introduction; highlights lack of systematic reviews on AI in ocular trauma. Page: 4–6
Objectives	4	Provide an explicit statement of the objective(s) or question(s) the review addresses.	Objectives are explicitly stated at the end of the Introduction. Page: 5–6
**METHODS**			
Eligibility criteria	5	Specify the inclusion and exclusion criteria for the review and how studies were grouped for the syntheses.	Eligibility criteria (inclusion/exclusion) are defined in the Methods section. Page: 7 Line: 141–145
Information sources	6	Specify all databases, registers, websites, organisations, reference lists and other sources searched or consulted to identify studies. Specify the date when each source was last searched or consulted.	PubMed, Scopus, and Web of Science were searched up to May 3, 2025. Page: 7 Line:140
Search strategy	7	Present the full search strategies for all databases, registers and websites, including any filters and limits used.	Full search strategy with Boolean operators is described in the Methods. Page: 8–10
Selection process	8	Specify the methods used to decide whether a study met the inclusion criteria of the review, including how many reviewers screened each record and each report retrieved, whether they worked independently, and if applicable, details of automation tools used in the process.	Two reviewers independently screened studies; PRISMA flow diagram is included. Page: 10–11 Line:
Data collection process	9	Specify the methods used to collect data from reports, including how many reviewers collected data from each report, whether they worked independently, any processes for obtaining or confirming data from study investigators, and if applicable, details of automation tools used in the process.	Data were independently extracted by two Researcher via a predesigned Excel sheet on the basis of the review protocol. Page: 10 Line: 172
Data items	10a	List and define all outcomes for which data were sought. Specify whether all results that were compatible with each outcome domain in each study were sought (e.g. for all measures, time points, analyses), and if not, the methods used to decide which results to collect.	Outcomes include model performance (accuracy, AUC, sensitivity, specificity, etc.). Page: 10 Line: 173–176
	10b	List and define all other variables for which data were sought (e.g. participant and intervention characteristics, funding sources). Describe any assumptions made about any missing or unclear information.	Other variables include study design, trauma type, AI model, input type, sample size, country, etc. Page: 10 Line: 173–176
Study risk of bias assessment	11	Specify the methods used to assess risk of bias in the included studies, including details of the tool(s) used, how many reviewers assessed each study and whether they worked independently, and if applicable, details of automation tools used in the process.	Risk of bias was assessed using the PROBAST tool by two independent reviewers. Page: 12 Line: 199–206
Effect measures	12	Specify for each outcome the effect measure(s) (e.g. risk ratio, mean difference) used in the synthesis or presentation of results.	Effect measures were model performance metrics: AUC, accuracy, precision, sensitivity, specificity. Page: 10–11 Line: 183–187
Synthesis methods	13a	Describe the processes used to decide which studies were eligible for each synthesis (e.g. tabulating the study intervention characteristics and comparing against the planned groups for each synthesis (item #5)).	Studies were grouped and described by AI model type and Trauma Types and Data Modalities. Page: 12–15
	13b	Describe any methods required to prepare the data for presentation or synthesis, such as handling of missing summary statistics, or data conversions.	No data conversions were needed; full data available from included studies.
	13c	Describe any methods used to tabulate or visually display results of individual studies and syntheses.	Results were tabulated in summary tables and explained in narrative format. Page: 12–15, 35–52
	13d	Describe any methods used to synthesize results and provide a rationale for the choice(s). If meta-analysis was performed, describe the model(s), method(s) to identify the presence and extent of statistical heterogeneity, and software package(s) used.	No meta-analysis conducted due to heterogeneity; narrative synthesis was used. Page: 10 Line: 178
	13e	Describe any methods used to explore possible causes of heterogeneity among study results (e.g. subgroup analysis, meta-regression).	No formal heterogeneity analysis was conducted. Page: 10 Line: 178
	13f	Describe any sensitivity analyses conducted to assess robustness of the synthesized results.	No sensitivity analysis was conducted. Page: 10 Line: 178
Reporting bias assessment	14	Describe any methods used to assess risk of bias due to missing results in a synthesis (arising from reporting biases).	Reporting bias was not formally assessed.
Certainty assessment	15	Describe any methods used to assess certainty (or confidence) in the body of evidence for an outcome.	Certainty of evidence was not assessed using tools like GRADE.
**RESULTS**			
Study selection	16a	Describe the results of the search and selection process, from the number of records identified in the search to the number of studies included in the review, ideally using a flow diagram.	Study selection process is presented with PRISMA flow diagram and counts. Page: 11
	16b	Cite studies that might appear to meet the inclusion criteria, but which were excluded, and explain why they were excluded.	Excluded studies and reasons for exclusion are listed. Page: 11 Line: 190–192
Study characteristics	17	Cite each included study and present its characteristics.	Characteristics of included studies are summarized in a table and narrative. Page: 12–15, 35–52
Risk of bias in studies	18	Present assessments of risk of bias for each included study.	Risk of bias results are reported in narrative and visual format. Page: 12, 30–34
Results of individual studies	19	For all outcomes, present, for each study: (a) summary statistics for each group (where appropriate) and (b) an effect estimate and its precision (e.g. confidence/credible interval), ideally using structured tables or plots.	Results for each study (metrics and AI models) are summarized in tables and narrative. Page: 12–15, 35–52
Results of syntheses	20a	For each synthesis, briefly summarise the characteristics and risk of bias among contributing studies.	Findings from grouped studies are summarized narratively. Page: 12–15
	20b	Present results of all statistical syntheses conducted. If meta-analysis was done, present for each the summary estimate and its precision (e.g. confidence/credible interval) and measures of statistical heterogeneity. If comparing groups, describe the direction of the effect.	No statistical synthesis or meta-analysis was conducted.
	20c	Present results of all investigations of possible causes of heterogeneity among study results.	No subgroup or heterogeneity analysis performed.
	20d	Present results of all sensitivity analyses conducted to assess the robustness of the synthesized results.	No sensitivity analyses performed.
Reporting biases	21	Present assessments of risk of bias due to missing results (arising from reporting biases) for each synthesis assessed.	No formal assessment of reporting bias was conducted.
Certainty of evidence	22	Present assessments of certainty (or confidence) in the body of evidence for each outcome assessed.	No formal assessment of certainty of evidence was performed.
**DISCUSSION**			
Discussion	23a	Provide a general interpretation of the results in the context of other evidence.	Overall findings interpreted in the context of existing literature. Page: 15–18
	23b	Discuss any limitations of the evidence included in the review.	Limitations of the included studies are discussed. Page: 18–19
	23c	Discuss any limitations of the review processes used.	Limitations of the review process (e.g., search scope) are described. Page: 19 Line: 363–368
	23d	Discuss implications of the results for practice, policy, and future research.	Implications for practice and future research are clearly outlined. Page: 19–20
**OTHER INFORMATION**			
Registration and protocol	24a	Provide registration information for the review, including register name and registration number, or state that the review was not registered.	Registered in PROSPERO: CRD420251043768. Page: 7–8 Line: 149–150
	24b	Indicate where the review protocol can be accessed, or state that a protocol was not prepared.	Protocol described in the Methods section Page: 7–8 Line:148–152
	24c	Describe and explain any amendments to information provided at registration or in the protocol.	No amendments were made after registration.
Support	25	Describe sources of financial or non-financial support for the review, and the role of the funders or sponsors in the review.	No financial or institutional support received.
Competing interests	26	Declare any competing interests of review authors.	Authors declared no competing interests.
Availability of data, code and other materials	27	Report which of the following are publicly available and where they can be found: template data collection forms; data extracted from included studies; data used for all analyses; analytic code; any other materials used in the review.	All data are from publicly available published studies.

**Table 2:** PROBAST questions for risk-of-bias assessment

**Participants**	1.1 Were appropriate data sources used, e.g., cohort, RCT, or nested case-control study data?
	1.2. Were all inclusions and exclusions of participants appropriate?
**Predictors**	2.1. Were predictors defined and assessed in a similar way for all participants?
	2.2. Were predictor assessments made without knowledge of outcome data?
	2.3. Are all predictors available at the time the model is intended to be used?
**Outcome**	3.1. Was the outcome determined appropriately?
	3.2. Was a prespecified or standard outcome definition used?
	3.3. Were predictors excluded from the outcome definition?
	3.4. Was the outcome defined and determined in a similar way for all participants?
	3.5. Was the outcome determined without knowledge of predictor information?
	3.6. Was the time interval between predictor assessment and outcome determination appropriate?
**Analysis**	4.1 Were there a reasonable number of participants with the outcome?
	4.2. Were continuous and categorical predictors handled appropriately?
	4.3 Were all enrolled participants included in the analysis?
	4.4. Were participants with missing data handled appropriately?
	4.5. Was selection of predictors based on univariable analysis avoided?
	4.6. Were complexities in the data (e.g., censoring, competing risks, sampling of control participants) accounted for appropriately?
	4.7. Were relevant model performance measures evaluated appropriately?
	4.8. Were model overfitting, underfitting, and optimism in model performance accounted for?
	4.9. Do predictors and their assigned weights in the final model correspond to the results from the reported multivariable analysis? *?*

**Table 3:** Risk-of-bias assessment of each study by PROBAST questions

Study	1.1	1.2	2.1	2.2	2.3	3.1	3.2	3.3	3.4	3.5	3.6	4.1	4.2	4.3	4.4	4.5	4.6	4.7	4.8	4.9	Overall risk
Bao (2023)	PY	Y	Y	NI	Y	Y	Y	Y	Y	NI	Y	Y	PY	PY	NI	Y	Y	PY	Y	Y	-
Choi (2021)	Y	PY	Y	Y	Y	Y	Y	Y	Y	Y	Y	NI	Y	Y	PY	Y	Y	Y	Y	Y	-
Gernandt (2024)	NI	NI	NI	NI	Y	Y	Y	Y	PY	NI	PY	PY	Y	Y	NI	Y	NI	Y	NI	Y	±
Li (2020)	Y	NI	Y	Y	Y	PY	Y	Y	Y	Y	PY	NI	Y	Y	PY	Y	Y	Y	PY	Y	±
Meng (2023)	Y	Y	Y	Y	PY	Y	Y	Y	Y	Y	Y	Y	PY	Y	PY	Y	Y	Y	Y	Y	-
Ming (2024)	PY	Y	Y	NI	Y	Y	Y	Y	Y	PN	NI	PY	NI	Y	Y	NI	NI	Y	PN	NI	+
Sarioglu (2012)	Y	Y	Y	NI	Y	Y	Y	PY	Y	PN	NI	Y	Y	Y	PY	NI	NI	Y	PY	NI	+
Shariati (2024)	PY	Y	Y	NI	PY	Y	PY	Y	Y	NI	PY	Y	Y	Y	PY	Y	Y	Y	PY	Y	-
Umapathy (2020)	Y	Y	Y	Y	PY	Y	Y	Y	Y	Y	PY	PY	Y	Y	Y	Y	Y	Y	Y	PY	-
Zhou (2023)	Y	Y	Y	NI	Y	Y	Y	Y	Y	NI	PY	Y	Y	Y	PY	Y	Y	Y	Y	Y	-

Abbreviations: **N**, not for low risk of bias; **n.a.**, not assessed; **NI**, no information for risk of bias assessment; **PN**, probably not for low risk of bias; **PY**, probably yes for low risk of bias; **Y**, yes for low risk of bias; **+**, indicated high risk of bias; **-**, indicated low risk of bias; **±**, indicated unclear risk of bias

**Table 4:**
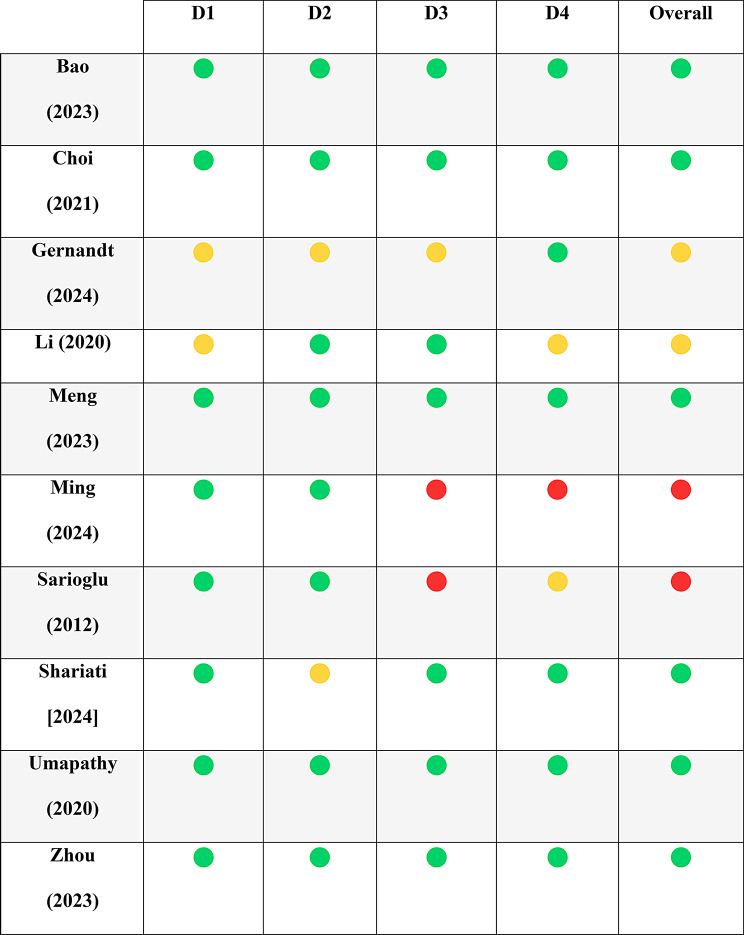
Risk of Bias assessment with color coding

**Table 5:** Data Extraction Table - Ocular Trauma & AI Studies

*N*	First Author, Year	Study Type	Trauma Type	AI Method	Input Data	Outcome Predicted	Sample Size	Country	Performance Metrics	Conclusion	Overall Risk of Bias (PROBAST)
1	Xiao-li Bao, 2023(20)	Development and internal validation	orbital blowout fractures (OBFs)	Deep learning networks (DenseNet-169 and UNet)	CT images	fracture side distinguishment and fracture area segmentation	3016 orbital CT images (497 cases)	China	**Evaluation of DenseNet-169 for Fracture Identification**: Accuracy:0.96 Sensitivity:0.97 Specificity:0.95 Precision:0.98 AUC:0.99 **Evaluation of DenseNet-169 for Fracture Side Distinguishment**: Accuracy:0.98 Sensitivity:0.97 Specificity:0.99 Precision:0.99 AUC:0.99	the developed AI system can automatically identify and segment orbital blowout fractures (OBFs). This advancement has the potential to enhance diagnostic accuracy and improve the efficiency of 3D printing-assisted surgical repairs for OBFs.	Low
2	Seungkwon Choi, 2021(18)	Retrospective Cohort Study	Open Globe Injuries (OGI)	9 Different algorithm	36 Clinical Features (e.g., retinal detachment, wound location, initial VA)	To predict final visual acuity and analyze significant factors influencing open globe injury prognosis. (Success: VA ≥ 0.1, Failure: VA < 0.1)	171 cases	Republic of Korea	**Support Vector Machine** Accuracy: 0.831 Precision: 0.856 Recall: 0.915 F Score: 0.884 AUC: 0.867 **Averaged Perceptron** Accuracy: 0.850 Precision: 0.881 Recall: 0.910 F Score: 0.894 AUC: 0.891 **Boosted Decision Tree** Accuracy: 0.899 Precision: 0.916 Recall: 0.944 F Score: 0.929 AUC: 0.971 **Bayes Point Machine** Accuracy: 0.855 Precision: 0.878 Recall: 0.930 F Score: 0.902 AUC: 0.902 **Decision Forest** Accuracy: 0.885 Precision: 0.914 Recall: 0.925 F Score: 0.919 AUC: 0.947 **Decision Jungle** Accuracy: 0.880 Precision: 0.912 Recall: 0.922 F Score: 0.916 AUC: 0.936 **Locally Deep Support Vector Machine** Accuracy: 0.852 Precision: 0.867 Recall: 0.937 F Score: 0.899 AUC: 0.887 **Logistic Regression** Accuracy: 0.841 Precision: 0.865 Recall: 0.922 F Score: 0.892 AUC: 0.893 **Neural Network** Accuracy: 0.844 Precision: 0.848 Recall: 0.948 F Score: 0.895 AUC: 0.898	The POTS model demonstrated high performance in predicting final visual acuity and was made available as a web-based tool.	Low
3	Steven Gernandt, 2024(14)	retrospective observational study	orbital fractures	ChatGPT-4	Age and gender of patient, Mechanism of injury, Current medical history, pertaining to the event, Relevant past medical history and comorbidities, Physical examination findings, Diagnostic imagery findings	assess the accuracy of ChatGPT-4 in the diagnosis and management of orbital fractures compared to the actual clinical decisions made	Unclear	Switzerland	**Diagnosis Accuracy** 100% (Correctly diagnosed fractures in all cases) **Management Discrepancy** 30% (Overall mismatch in treatment choice: conservative vs. surgical) **Specificity** 100% (Perfect identification of patients needing surgery) **Sensitivity** 57% (Moderate performance in identifying cases for conservative management0 **Positive Predictive Value (PPV)** 100% (All predicted surgical cases truly needed surgery) **Negative Predictive Value (NPV)** 50% (Only half of predicted conservative cases truly suited it)	The ChatGPT-4 model can be a useful tool in the diagnosis and management of orbital fractures; however, further studies are needed to evaluate its generalizability and clinical applicability.	Moderate
4	Lunhao Li, 2020(17)	Retrospective Cohort Study	orbital factures	Inception V3 (CNN) + XGBoost convolutional neural networks	Orbital CT scans	Detection of Orbital Fractures	188 CT scans (94 fracture, 94 control	China	Accuracy: 92% (single-image), 87% (patient-level), AUC: 0.957	The model showed good performance (high AUC), but external validation with multicenter data is needed.	Moderate
5	Xiangda Meng, 2023(21)	Multicenter Retrospective Cohort + Prospective Validation	open globe injury (OGI)-no light perception (NLP)	Machine Learning (XGBoost with Nested Cross-Validation)	16 Clinical Features (e.g., wound length, vitreous status, retinal detachment)	predict the visual outcomes of vitrectomy for OGI-NLP eyes (Visual Recovery (NLP vs. Light Perception/Improved Vision))	531 Patients (459 Training + 72 External Validation + 27 Prospective)	China	AUC: 0.75 (Pre-Vitrectomy), 0.90 (Intra-Vitrectomy)	The VisionGo model outperformed the traditional OTS system and ophthalmologists, and offered high interpretability using SHAP.	Low
6	Shuai Ming, 2024(22)	A cross-sectional study	Eye disease	Artificial intelligence (AI) chatbots (chat GPT)	Medical history (Hx) + examination findings (Hx + Ex) in textual format	investigate the performance of AI chatbots in recommending ophthalmic outpatient registration and diagnosing eye diseases within clinical case profiles.	208 cases	China	**Registration Suggestion Accuracy (Hx profiles only)**: **GPT-3.5**:66/104 → **63.5%** **GPT-4.0**: 81/104 → **77.9%** **Residents**: 72/104 → **69.2%** **P value**: 0.07 **Top-performing subspecialties**: Ocular trauma, retinal diseases, and strabismus/amblyopia.	ChatGPT (especially GPT-4.0) showed moderate performance in residency recommendations and disease diagnosis, but further validation is needed for clinical use	High
7	Efsun Sarioglu, 2012(23)	Retrospective	traumatic orbital injury	Natural Language Processing (NLP) with MedLEE + Topic Modeling (LDA)	Emergency CT scan textual reports	Diagnosis of orbital fracture (positive/negative)	3705 cases (463 positive, 3,242 negative)	USA	**CI:95%** **Decision Tree** Precision: 0.947 Recall: 0.948 F Score: 0.948 **NLP - All**: Precision: 0.955 Recall: 0.956 F Score: 0.955 **NLP - Filtered**: Precision: 0.970 Recall: 0.970 F Score: 0.970 **SVM** Precision: 0.959 Recall: 0.960 F Score: 0.959 **NLP - All**: Precision: 0.968 Recall: 0.969 F Score: 0.968 **NLP - Filtered**: Precision: 0.971 Recall: 0.971 F Score: 0.971	NLP with UMLS code filtering showed the best performance. Topic modeling was useful for dimensionality reduction, but binary classification performed poorly.	High
8	Mehrdad Motamed Shariati, 2024(24)	Analytical-observational (retrospective)	Open Globe Injuries (OGI)	Different machine learning models (MLR, SVM, KNN, DT, RF, ADA, BG, WGB, ANN, PPV, BC, MCV)	12clinical features (age, gender, type of trauma, initial VA, RAPD, injury zone, ocular complications such as RD, TON, IOFB, etc.)	Final visual acuity (VA) in three classes: poor (0–0.1), moderate (0.1–0.7), good (0.7–1)	301 cases	Iran	**CI:95%** **SVM** AUC-ROC: 0.86 AUC-PRC: 0.75 PPV: 0.812 Sensitivity: 0.764 Accuracy: 0.885 F1 Score: 0.787 MCC: 0.709 Brier Score (BS): 0.433	The ANN model showed the best performance (AUC-ROC: 0.96, Accuracy: 93%)	Low
									**Naïve Bayes** AUC-ROC: 0.84 AUC-PRC: 0.72 PPV: 0.857 Sensitivity: 0.705 Accuracy: 0.885 F1 Score: 0.774 MCC: 0.704 Brier Score (BS): 0.634		
									**XGB** AUC-ROC: 0.88 AUC-PRC: 0.82 PPV: 0.923 Sensitivity: 0.705 Accuracy: 0.901 F1 Score: 0.800 MCC: 0.744 Brier Score (BS): 0.395		
									**ADA** AUC-ROC: 0.86 AUC-PRC: 0.74 PPV: 0.750 Sensitivity: 0.705 Accuracy: 0.852 F1 Score: 0.727 MCC: 0.626 Brier Score (BS): 0.643		
									**Bagging** AUC-ROC: 0.89 AUC-PRC: 0.79 PPV: 0.764 Sensitivity: 0.764 Accuracy: 0.868 F1 Score: 0.764 MCC: 0.673 Brier Score (BS): 0.412		
									**Multinomial Logistic Regression** AUC-ROC: 0.84 AUC-PRC: 0.75 PPV: 0.866 Sensitivity: 0.764 Accuracy: 0.901 F1 Score: 0.812 MCC: 0.748 Brier Score (BS): 0.439		
									**KNN** AUC-ROC: 0.81 AUC-PRC: 0.69 PPV: 0.750 Sensitivity: 0.705 Accuracy: 0.852 F1 Score: 0.727 MCC: 0.626 Brier Score (BS): 0.476		
									**Decision Tree** AUC-ROC: 0.85 AUC-PRC: 0.75 PPV: 0.785 Sensitivity: 0.647 Accuracy: 0.852 F1 Score: 0.709 MCC: 0.617 Brier Score (BS): 0.413		
									**Random Forest (RF)** AUC-ROC: 0.92 AUC-PRC: 0.86 PPV: 0.866 Sensitivity: 0.764 Accuracy: 0.901 F1 Score: 0.812 MCC: 0.748 Brier Score (BS): 0.311		
									**Artificial Neural Network (ANN)** AUC-ROC: 0.96 AUC-PRC: 0.91 PPV: 0.894 Sensitivity: 0.819 Accuracy: 0.930 F1 Score: 0.855 MCC: 0.811 Brier Score (BS): 0.201		
9	L Umapathy, 2020(25)	Retrospective	Ocular trauma (specifically globe injuries)	Deep learning using a 2D Modified Residual UNET (MRes-UNET2D)	CT images	Automated segmentation of globe contours and volume quantification	Training on 80 subjects without globe injuries; testing on two cohorts: 18 subjects in the first cohort and 9 subjects in the second cohort	USA	**First Test Cohort 9n = 18)** Dice Score: 0.95 Precision: 96% Recall: 95% **Second Test Cohort** (*n* = 9) MRes-UNET2D Dice Score: 0.95 Human Interobserver Dice Score: 0.94	The MRes-UNET2D model enables fast and reliable segmentation and volume estimation of globes in orbital CT images. Its performance is comparable to human experts, and it can serve as a quantitative tool in ocular trauma assessment.	Low
10	Zhi-Lu Zhou, 2023(26)	retrospective analysis experiment	ocular trauma	machine learning models (XGB, SVR, BYR, RFR)	clinical data, OCT images and fundus photographs	assessment of VA after ocular trauma	100 eyes	China	**CI:95%** Sensitivity: 0.83 Precision: 0.92 Specificity: 0.95 Accuracy: 0.90	Predicting BCVA using machine-learning models in patients with treated ocular trauma is accurate and helpful in the identification of visual dysfunction.	Low

## Data Availability

No datasets were generated or analysed during the current study.

## Abbreviations

AI: Artificial Intelligence
ANN: Artificial Neural Network
AUC: Area Under the Curve
BCVA: Best Corrected Visual Acuity
CNN: Convolutional Neural Network
CT: Computed Tomography
GPT: Generative Pre-trained Transformer
IOFB: Intraocular Foreign Body
MCC: Matthews Correlation Coefficient
ML: Machine Learning
NLP: Natural Language Processing (یا No Light Perception - با توجه به context)
OBF: Orbital Blowout Fracture
OGI: Open Globe Injury
OCT: Optical Coherence Tomography
OTS: Ocular Trauma Score
PRISMA: Preferred Reporting Items for Systematic Reviews and Meta-Analyses
PROBAST: Prediction model Risk Of Bias ASsessment Tool
RAPD: Relative Afferent Pupillary Defect
RCT: Randomized Controlled Trial
RD: Retinal Detachment
SHAP: Shapley Additive Explanations
SVM: Support Vector Machine
TON: Traumatic Optic Neuropathy
VA: Visual Acuity
XGB: Extreme Gradient Boosting
XGBoost: Extreme Gradient Boosting
2D: Two-Dimensional
3D: Three-Dimensional

## References

[1] Messman AM. Ocular Injuries: New Strategies In Emergency Department Management. Emerg Med Pract. 2015;17(11):1–21. quiz. 26466300

[2] Gervasio KA, Weinstock BM, Wu AY. Prognostic value of ocular trauma scores in patients with combined open globe injuries and facial fractures. Am J Ophthalmol. 2015;160(5):882–8. e2. 26275473 10.1016/j.ajo.2015.08.007

[3] Yan H. Problems and countermeasures of ocular trauma emergency management in China. [Zhonghua yan ke za Zhi]. Chin J Ophthalmol. 2019;55(9):641–4. 10.3760/cma.j.issn.0412-4081.2019.09.001 31495148

[4] Iserson KV, Moskop JC. Triage in medicine, part I: concept, history, and types. Ann Emerg Med. 2007;49(3):275–81. 17141139 10.1016/j.annemergmed.2006.05.019

[5] Lindfield D, Das-Bhaumik R. Emergency department management of penetrating eye injuries. Int Emerg Nurs. 2009;17(3):155–60. 19577202 10.1016/j.ienj.2009.01.003

[6] Khare G, Andrew Symons R, Do D. Common ophthalmic emergencies. Int J Clin Pract. 2008;62(11):1776–84. 19143862 10.1111/j.1742-1241.2008.01855.x

[7] Resnikoff S, Felch W, Gauthier T-M, Spivey B. The number of ophthalmologists in practice and training worldwide: a growing gap despite more than 200 000 practitioners. Br J Ophthalmol. 2012;96(6):783–7. 22452836 10.1136/bjophthalmol-2011-301378

[8] Beshay N, Keay L, Dunn H, Kamalden TA, Hoskin AK, Watson SL. The epidemiology of Open Globe Injuries presenting to a tertiary referral eye hospital in Australia. Injury. 2017;48(7):1348–54. 28438416 10.1016/j.injury.2017.04.035

[9] Négrel A-D, Thylefors B. The global impact of eye injuries. Ophthalmic Epidemiol. 1998;5(3):143–69. 9805347 10.1076/opep.5.3.143.8364

[10] McGwin G Jr., Owsley C. Incidence of emergency department-treated eye injury in the United States. Arch Ophthalmol. 2005;123(5):662–6. 15883286 10.1001/archopht.123.5.662

[11] Anyoha R. The history of artificial intelligence. Sci News. 2017;28.

[12] Amisha MP, Pathania M, Rathaur V. Overview of artificial intelligence in medicine. J Family Med Prim Care. 2019;8(7):2328–31. 31463251 10.4103/jfmpc.jfmpc_440_19 PMC6691444

[13] Chen S-C, Chiu H-W, Chen C-C, Woung L-C, Lo C-M. A novel machine learning algorithm to automatically predict visual outcomes in intravitreal ranibizumab-treated patients with diabetic macular edema. J Clin Med. 2018;7(12):475. 30477203 10.3390/jcm7120475 PMC6306861

[14] Gernandt S, Aymon R, Scolozzi P. Assessing the accuracy of artificial intelligence in the diagnosis and management of orbital fractures: Is this the future of surgical decision-making? JPRAS Open. 2024;42:275–83. 39498287 10.1016/j.jpra.2024.09.014 PMC11532732

[15] Du X-L, Li W-B, Hu B-J. Application of artificial intelligence in ophthalmology. Int J Ophthalmol. 2018;11(9):1555. 30225234 10.18240/ijo.2018.09.21 PMC6133903

[16] Labib KM, Ghumman H, Jain S, Jarstad JS. Applications of Artificial Intelligence in Ophthalmology: Glaucoma, Cornea, and Oculoplastics. Cureus. 2024;16(11):e73522. 39677277 10.7759/cureus.73522 PMC11638466

[17] Li L, Song X, Guo Y, Liu Y, Sun R, Zou H, et al. Deep Convolutional Neural Networks for Automatic Detection of Orbital Blowout Fractures. J Craniofac Surg. 2020;31(2):400–3. 31842071 10.1097/SCS.0000000000006069

[18] Choi S, Park J, Park S, Byon I, Choi HY. Establishment of a prediction tool for ocular trauma patients with machine learning algorithm. Int J Ophthalmol. 2021;14(12):1941–9. 34926212 10.18240/ijo.2021.12.20 PMC8640771

[19] Page MJ, McKenzie JE, Bossuyt PM, Boutron I, Hoffmann TC, Mulrow CD, et al. The PRISMA 2020 statement: an updated guideline for reporting systematic reviews. BMJ. 2021;372:n71. 33782057 10.1136/bmj.n71 PMC8005924

[20] Bao XL, Zhan X, Wang L, Zhu Q, Fan B, Li GY. Automatic identification and segmentation of orbital blowout fractures based on Artificial Intelligence. Translational Vis Sci Technol. 2023;12(4). 10.1167/tvst.12.4.7 PMC10082383 37022710

[21] Meng X, Wang Q, Chen S, Zhang S, Yu J, Li H, et al. An interpretable model predicts visual outcomes of no light perception eyes after open globe injury. Br J Ophthalmol. 2023;108(2):285–93. 10.1136/bjo-2022-322753 36596662

[22] Ming S, Guo XH, Guo QG, Xie KP, Chen DD, Lei B. Performance of ChatGPT in Ophthalmic Registration and ClinicalDiagnosis:Cross-Sectional Study. J Med Internet Res. 2024;26:14. 10.2196/60226 PMC11605262 39541581

[23] Sarioglu E, Choi HA, Yadav K. Clinical report classification using Natural Language Processing and Topic Modeling. Proc Int Conf Mach Learn Appl. 2012;2012:204–9. 37767274 10.1109/icmla.2012.173 PMC10530625

[24] Shariati MM, Eslami S, Shoeibi N, Eslampoor A, Sedaghat M, Gharaei H et al. Development, comparison, and internal validation of prediction models to determine the visual prognosis of patients with open globe injuries using machine learning approaches. BMC Med Inf Decis Mak. 2024;24(1). 10.1186/s12911-024-02520-4 PMC11106970 38773484

[25] Umapathy L, Winegar B, MacKinnon L, Hill M, Altbach MI, Miller JM, et al. Fully automated segmentation of globes for volume quantification in CT images of orbits using deep learning. Am J Neuroradiol. 2020;41(6):1061–9. 32439637 10.3174/ajnr.A6538 PMC7342761

[26] Zhou ZL, Yan YF, Chen JM, Liu RJ, Yu XY, Wang M, et al. Predicting visual acuity with machine learning in treated ocular trauma patients. Int J Ophthalmol. 2023;16(7):1005–14. 37465511 10.18240/ijo.2023.07.02 PMC10333235

[27] Wang S, He X, Jian Z, Li J, Xu C, Chen Y, et al. Advances and prospects of multi-modal ophthalmic artificial intelligence based on deep learning: a review. Eye Vis. 2024;11(1):38. 10.1186/s40662-024-00405-1 PMC11443922 39350240

[28] Ong AY, Taribagil P, Sevgi M, Kale AU, Dow ER, Macdonald T, et al. A scoping review of artificial intelligence as a medical device for ophthalmic image analysis in Europe, Australia and America. npj Digit Med. 2025;8(1):323. 40442400 10.1038/s41746-025-01726-8 PMC12122805

[29] Sorin V, Kapelushnik N, Hecht I, Zloto O, Glicksberg BS, Bufman H, et al. Integrated visual and text-based analysis of ophthalmology clinical cases using a large language model. Sci Rep. 2025;15(1):4999. 39930078 10.1038/s41598-025-88948-8 PMC11811221

[30] Nayagam G, Raman M, Anuradha A, Sheela S, Chakravarthy N. Assessment of Visual Prognosis Using Ocular Trauma Score in Open Globe Injury at a Tertiary Care Center. TNOA J Ophthalmic Sci Res. 2020;58(1):9–13.

[31] Man C, Steel D. Visual outcome after open globe injury: a comparison of two prognostic models—the Ocular Trauma Score and the Classification and Regression Tree. Eye. 2010;24(1):84–9. 19229267 10.1038/eye.2009.16

[32] Ojaghihaghighi S, Lombardi KM, Davis S, Vahdati SS, Sorkhabi R, Pourmand A. Diagnosis of Traumatic Eye Injuries With Point-of-Care Ocular Ultrasonography in the Emergency Department. Ann Emerg Med. 2019;74(3):365–71. 30905470 10.1016/j.annemergmed.2019.02.001

[33] Vrablik ME, Snead GR, Minnigan HJ, Kirschner JM, Emmett TW, Seupaul RA. The diagnostic accuracy of bedside ocular ultrasonography for the diagnosis of retinal detachment: a systematic review and meta-analysis. Ann Emerg Med. 2015;65(2):199–e2031. 24680547 10.1016/j.annemergmed.2014.02.020

[34] Warin K, Limprasert W, Suebnukarn S, Paipongna T, Jantana P, Vicharueang S. Maxillofacial fracture detection and classification in computed tomography images using convolutional neural network-based models. Sci Rep. 2023;13(1):3434. 36859660 10.1038/s41598-023-30640-w PMC9978019

[35] Dashti M, Ghaedsharaf S, Ghasemi S, Zare N, Constantin EF, Fahimipour A, et al. Evaluation of deep learning and convolutional neural network algorithms for mandibular fracture detection using radiographic images: A systematic review and meta-analysis. Imaging Sci Dent. 2024;54(3):232–9. 39371302 10.5624/isd.20240038 PMC11450407

[36] Lundberg SM, Lee S-I. A unified approach to interpreting model predictions. Adv Neural Inf Process Syst. 2017;30.

[37] Tan TF, Dai P, Zhang X, Jin L, Poh S, Hong D, et al. Explainable artificial intelligence in ophthalmology. Curr Opin Ophthalmol. 2023;34(5):422–30. 37527200 10.1097/ICU.0000000000000983

